# [Corrigendum] TERT promoter regulating melittin expression induces apoptosis and G_0_/G_1_ cell cycle arrest in esophageal carcinoma cells

**DOI:** 10.3892/ol.2026.15651

**Published:** 2026-05-15

**Authors:** Chao Zhou, Jie Ma, Yuanhua Lu, Wan Zhao, Bingxue Xu, Jian Lin, Yongjun Ma, Yafei Tian, Qi Zhang, Wei Wang, Weiqun Yan, Ping Jiao

Oncol Lett 21: 16, 2021; DOI: 10.3892/ol.2020.12277

Subsequently to the publication of the above article, an interested reader drew to the authors’ attention that, concerning the cellular images shown in Fig. 3A on p. 5, the ‘Blank’ and ‘Con’ data panels showed an overlapping section, such that these were apparently derived from the same original source where the results of differently performed experiments were intended to have been portrayed.

After re-examining their original data, the authors have realized that some of the data in Fig. 3A were inadvertently assembled incorrectly. The revised version of Fig. 3, now showing the correct data for the ‘Blank’ panel in Fig. 3A, is shown on the next page. The authors are grateful to the Editor of *Oncology Letters* for allowing them this opportunity to publish a Corrigendum, and all the authors agree with its publication. Furthermore, the authors apologize to the readership for any inconvenience caused.

## Figures and Tables

**Figure 3.**
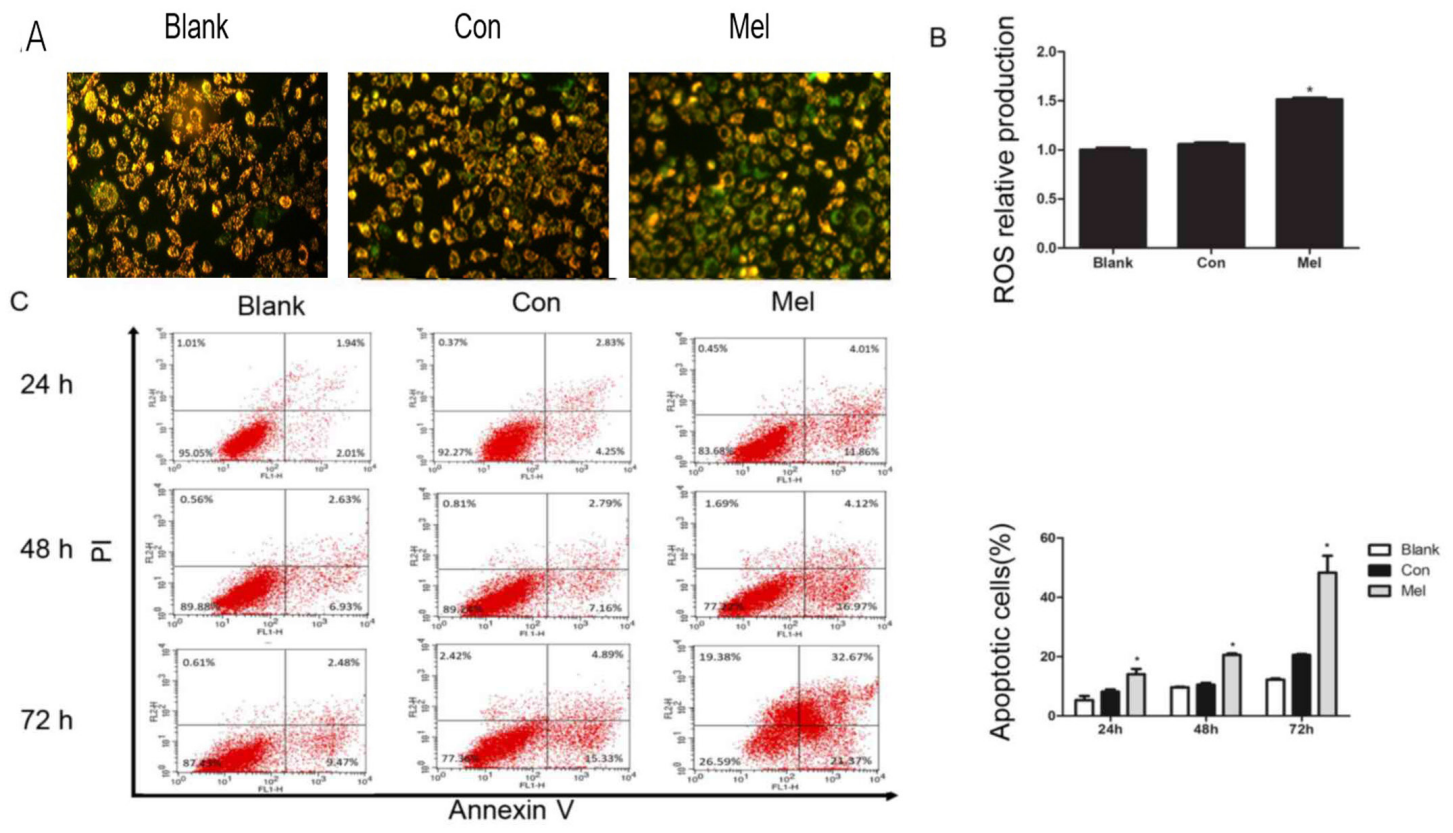
Transfection of pcTERT-Mel decreases mitochondrial membrane potential and increases ROS production in TE1 cells, leading to apoptosis. (A) Cells were stained with tetraethylbenzimidazolylcarbocyanine iodide and visualized using a fluorescence microscope at 24 h post-transfection. pcTERT treated cells and untreated cells stained red suggested normal high membrane potentials. pcTERT-Mel treatment caused a significant loss of red fluorescence and an increase of green fluorescence, indicating the loss of mitochondrial membrane potential, which was associated with apoptosis (original magnification, ×200). (B) ROS production was detected with a ROS assay kit. Increased ROS production was observed in pcTERT-melittin treated cells with a fluorescence microplate at excitation and emission wavelengths of 488 and 525 nm, respectively. (C) Quantification of the pcTERT-melittin transfection-induced apoptosis of TE1 cells, as assessed via flow cytometry using Annexin-V and PI staining at 24, 48 and 72 h post-transfection. The percentage of apoptotic cells was presented as the mean ± SEM. Results are an average of three independent experiments. *P<0.05 vs. Con group. TERT, telomerase reverse transcriptase; Con, control; Mel, melittin; ROS, reactive oxygen species.

